# Quantifying structural selection bias in observational cohort data: a ponderation analysis of age - specific incidence rates to inform vaccine safety verification

**DOI:** 10.3389/fphar.2025.1754809

**Published:** 2026-01-08

**Authors:** Marco Roccetti

**Affiliations:** Department of Computer Science and Engineering, University of Bologna, Bologna, Italy

**Keywords:** COVID-19 vaccine safety, detection bias, direct age-standardization analysis, Hazard ratio decomposition, internal/external validity, structural selection bias

## Abstract

**Background:**

A recent nationwide cohort study reported an unadjusted Hazard Ratio (HR) of 2.714 for Vitiligo incidence following COVID-19 vaccination, indicating a major safety concern. This finding was based on cohorts with an ≈ 11-year age difference, immediately raising critical concerns regarding extreme structural selection and detection bias.

**Objectives:**

We hypothesize that this extreme association is an artifact of a fatal methodological flaw, challenging the study’s internal validity and subsequent external validity. We aim to quantitatively separate the HR attributable to the structural age imbalance (HR Structural) from the residual HR (HR Residual), which quantifies the uncorrected methodological failure and residual confounding. We further perform a plausible recalculation of risk to demonstrate the complete collapse of the risk signal upon correcting the methodological failure in the baseline cohort.

**Methods:**

We performed a direct age-standardization analysis analysis using the age distribution of the scrutinized study’s cohorts (Vaccinated, mean age = 56.32 years vs. Non-Vaccinated, mean age = 45.51 years) and applied established national age-specific Vitiligo incidence rates (IR) from external epidemiology.

**Results:**

The HR Structural was calculated to be 1.2104. The remaining HR Residual of 2.2423 quantifies the uncorrected methodological failure. The NV cohort’s observed incidence rate (0.67/10,000) was found to be nearly 70% lower than the expected rate (2.2146/10,000), providing quantifiable evidence of profound non-comparability. The subsequent recalculation of risk, correcting for this baseline failure, reduces the observed HR of = 2.714 to an HR Corrected of 1.0025, thus completely annulling the signal of risk due to vaccination.

**Discussion:**

The HR = 2.714 of the scrutinized study is an unstable statistical artifact. The overwhelming majority of the observed association is a consequence of a fatal design flaw. The HR Corrected of almost 1 confirms that correcting the methodological error eliminates the risk signal, demonstrating a severe lack of internal and external validity of the original study.

## 1 Introduction

Observational studies utilizing national registries, such as those conducted in South Korea (Kim et al., 2024), represent a critical resource for post-marketing surveillance and vaccine safety verification. However, the reliance on pre-existing data necessitates strict adherence to established methodological standards, notably the STROBE (STrengthening the Reporting of Observational studies in Epidemiology) guidelines (Elm et al., 2007). The primary goal is to ensure internal validity, that the observed association is real within the study context, which is a prerequisite for achieving external validity (that is generalizability to the broader population).

The recent study published in Kim et al. (2024) reported a strikingly high, unadjusted Hazard Ratio of 2.714 for Vitiligo following COVID-19 vaccination, based on a comparison between a Vaccinated (V) cohort (mean age = 56.32 years) and a Non-Vaccinated (Non-Vaccinated) cohort (mean age = 45.51 years). This ≈11-year age difference immediately flagged critical concerns regarding confounding by indication and immortal time bias (Terao, 2025). The sheer magnitude of the ≈11-year age difference, coupled with the cumulative incidence rates observed (2.22 vs. 0.67 per 10,000), strongly suggests that the cohorts were inherently non-comparable.

Our analysis posits that the reported HR = 2.714 is not a reflection of a robust biological signal but rather a quantitative measure of a fatal design flaw. We hypothesize that an Extreme Structural Selection and Detection Bias was introduced by defining the cohorts in a manner that artificially minimized the baseline risk in the NV group, while simultaneously maximizing the detection and prevalence risk in the V group. We present a rigorous, quantitative method, that is a stratified ponderation analysis, or better said a direct age-standardization analysis, using external South Korean national age-specific incidence data, to decompose the observed HR and isolate the true contribution of the structural bias (Lee et al., 2015; Sun et al., 2021; Chemaitelly et al., 2025; Roccetti and Cacciapuoti, 2025; Jones and Podolsky, 2015).

The quantitative findings of the present research confirm this hypothesis: the structural age difference alone accounts for a calculated Structural Hazard Ratio (HR Structural) of 1.2104. Importantly, we demonstrate that the non-comparability of the baseline cohort (NV) caused its observed incidence rate to drop nearly 70% below the expected rate. The overwhelming majority of the observed association is captured by the residual Hazard Ratio (HR Residual of 2.2423) which stands as a clear measure of the uncorrected methodological failure. We further show that applying a reasonable recalculation of risk, which corrects the failure of the baseline incidence rate, reduces the observed risk signal from 2.714 to a clinically insignificant HR of approxately 1 (HR Corrected).

Essentially, the quantitative findings of the present research has moved along two concatenated directions. First we confirm this hypothesis that the structural age difference alone accounts for a calculated structural Hazard Ratio (HR Structural) of 1.2104. This means that the observed demographic imbalance represents an increase of about one-fifth of the reported excess risk based on demographics alone. Then, we show that the majority of the association captured by the residual Hazard Ratio (HR Residual) of 2.2423 simply stands as a clear measure of the uncorrected methodological failure and unmeasured confounding. This substantial residual value not only strongly indicates that the cohorts were not subject to a common support, leading to a profound violation of the assumption of comparability required by the Cox Proportional Hazards model but can be also explained if one takes into account the drop of nearly 70% in the incidence rate of Vitiligo in the NV group. Putting this information into the calculation easily leads to a corrected value of HR of almost 1 that practically annuls the risk difference between V and NV subgroups.

Ultimately, the goal of this re-evaluation is to reassert the imperative for epidemiological validity in studies of vaccine safety derived from observational data. We demonstrate that sophisticated statistical adjustments cannot remedy fundamental flaws in cohort design where non-measured confounding factors, such as health-seeking behavior, surveillance frequency and distribution of the disease incidence peaks are unevenly distributed (Sun et al., 2021). By quantitatively isolating and measuring the non-causal structural bias, our analysis provides a critical framework for interpreting extreme risk estimates and ensuring that public health conclusions are based on associations that are epidemiologically sound, rather than artifactual.

## 2 Methods

We here provide all the fundamental methods and data useful for the aim of pondering the structural bias on which we are investigating.

### 2.1 Study data and baseline characteristics

We extracted the following key data from Kim et al. (2024) to establish the basis of the structural bias as reported in the following Table 1.

**TABLE 1:** Baseline characteristics and unadjusted incidence rates from Kim et al. (2024).

Cohort	Mean age	Standard deviation (SD)	Cumulative incidence rate (at 3 months) (per 10,000 p-y)
Non-Vaccinated (NV)	45.51 years	17.31	P(NV) = 0.67
Vaccinated (V)	56.32 years	16.55	P(V) = 2.22

### 2.2 Direct age-standardization analysis

We performed a first preliminary quantitative analysis by combining the age distribution percentages P(i) of the V and NV groups of Kim et al. (2024) with independent, established age-specific annual incidence rates IR(i) for Vitiligo in South Korea, based on 2019 data as reported in Kang and Lee (2023). It is reported in Table 2 below.

**TABLE 2:** Input data for direct age-standardization analysis.

Age group	South Korean IR (per 10,000 p-y)	% In NV group P (i, NV)	% In V group P (i,V)
<20 years	3.4241	Not included in Kim et al. (2024)	Not included in Kim et al. (2024)
20–29 years	1.5717	18.46%	9.92%
30–39 years	1.7813	25.49%	7.70%
40–49 years	1.9053	20.92%	10.82%
50–59 years	2.5874	14.16%	24.76%
≥ 60 years	3.3643	20.97%	45.79%
Total	—	100%	100%

We selected the 2019 incidence data (Kang and Lee, 2023) as the external reference rate because it represents the most recent pre-pandemic and pre-vaccination baseline available. This choice ensures that the calculated expected rates IR Expected are not confounded by systemic changes in prevalence or healthcare utilization introduced by the COVID-19 era. Furthermore, the robustness of this specific national incidence data (Kang and Lee, 2023) is confirmed by the already-cited 2024 Lancet study (Akl et al., 2024).

The external rates utilized are derived from the same South Korean National Health Insurance Service (NHIS) database employed by the scrutinized study (Kim et al., 2024), guaranteeing high methodological comparability, as both studies draw from the identical national surveillance system. The age categories used for the direct age-standardization analysis (Table 2) are directly mapped from the reporting strata provided in the source of the external incidence data and the demographic breakdowns available in the scrutinized study, ensuring maximum fidelity to the published sources.

A key observation is that, it should be noted here that the composition of the NV group is strongly influenced by the presence of young individuals who are already past the first peak of Vitiligo onset, contrasting with the massive presence in the NV group of older individuals entirely included in the interval where the second incidence peak of Vitiligo manifests, which is universally recognized as having a bimodal distribution (under 20, over 40/50 years) (Kang and Lee, 2023).

The age categories used for the direct age-standardization analysis (Table 2) are directly mapped from the reporting strata provided in the source of the external incidence data and the demographic breakdowns available in the scrutinized study (Kim et al., 2024), ensuring maximum fidelity to the published sources.This mandatory exclusion of the high-risk <20 age stratum, a population known to exhibit a significant incidence peak confirmed by clinical practice and literature (Akl et al., 2024; Kang and Lee, 2023), highlights a crucial methodological failing by the original authors in defining a truly representative reference cohort, with the responsibility for incorporating or accurately matching such known structural risk factors falls solely upon the study design. For methodological integrity and in deference to the authors of Kim et al. (2024) we have also assumed the incidence rates reported in the source study to represent harmonically annualized figures, consistent with standard epidemiological practice, rather than extrapolating quarterly rates.

### 2.3 Calculation of HR structural and HR residual

The Expected Annual Incidence Rate, IR (Expected) for each cohort, based solely on its structural age composition, can be calculated using the following Formula 1:

$$\text{IR}\left(\text{Expected}\right)=\sum \left(\text{IR}\left(i\right)\times P\left(i\right)\right)$$ (1)

where IR(i) are the South Korean age-specific incidence rates from external data (Table 2) and P(i) are the proportional distributions of the respective cohort (V or NV) reported in the same Table 2.

Applying this age-standardization to the Non-Vaccinated (NV) cohort demographics, we obtain the baseline expected incidence, IR (NV, Expected) exactly as follows:

IR (NV, Expected) = (1.5717 × 0.1846) + (1.7813 × 0.2549) + (1.9053 × 0.2092) + (2.5874 × 0.1416) + (3.3643 × 0.2097) ≈ 2.2146/10,000.

Similarly, applying this age-standardization to the Vaccinated (V) cohort demographics yields IR (V, Expected):

IR (V, Expected) = (1.5717 × 0.0992) + (1.7813 × 0.0770) + (1.9053 × 0.1082) + (2.5874 × 0.2476) + (3.3643 × 0.4579) ≈ 2.6804/10,000.

This allowed us to calculate the HR Structural as follows: HR Structural = 2.6804/2.2146 ≈ 1.2104. While HR Structural is mathematically equivalent to an Incidence Rate Ratio, we maintain the HR nomenclature for consistency with the final reported metric of the scrutinized study (HR Observed), a convention that is epidemiologically acceptable given the rarity of the outcome.

Finally, the HR Residual can be computed as the ratio between the HR provided in Kim et al. (2024) (termed HR Observed) and our computed HR Structural: HR Residual = HR Observed/HR Structural = 2.714/1.2104 ≈ 2.2423.

The consistency of this multiplicative decomposition is confirmed by the near-exact reconstruction of the observed value: HR Structural × HR Residual ≈1.2104 × 2.2423 ≈ 2.714.

## 3 Results

We here provide the two main set of results stemming from our analysis whose methodology has been anticipated in the previous Section.

### 3.1 Decomposition of the observed Hazard Ratio and incidence discrepancy

Following the calculation of the Expected Incidence Rates IR (Expected) based solely on the structural age compositions of the two cohorts (Section 2.3), we proceeded to quantify the true extent of the methodological failure. This involved decomposing the high, observed HR Observed = 2.714 from Kim et al. (2024) into two distinct components: the risk attributable purely to the structural age imbalance (HR Structural) and the risk stemming from all other uncorrected design flaws and selection biases (HR Residual). Since Hazard Ratios combine *multiplicatively*, that is HR Observed = HR Structural × HR Residual, the HR Residual thus acts as a precise metric for the degree of non-comparability that persists despite accounting for the known age difference. The breakdown of this risk is definitely presented in Table 3.

**TABLE 3:** Decomposing the observed Hazard ratio [HR = 2.714 (Kim et al., 2024)].

Parameter	Description	Value	Contribution to excess risk: (HR_component_ −1)/(HR_Observed_ −1) x 100
HR Observed	Unadjusted Hazard Ratio from Kim et al. (2024)	2.714	100%
HR Structural	HR due to Age Structural Bias Alone	1.2104	12.28%
HR Residual	HR Unexplained by Structural Age Bias	2.2423	87.72%

The most salient finding of the direct age-standardization analysis shown above is the extreme divergence between the expected and observed incidence rates in the reference cohort. The demographic composition of the Non-Vaccinated NV subgroup predicted an expected Incidence Rate of 2.2146/10,000 based on established national age-specific rates. However, the rate observed in Kim et al. (2024) was only 0.67/10,000. This discrepancy means the observed incidence in the reference cohort was nearly 70% lower than methodologically expected, providing immediate, quantifiable evidence of the profound non-comparability of the baseline groups.

This conclusion remains robust even if one were to hypothesize that the 0.67/10,000$ figure in Kim et al. (2024) represented a quarterly rate, which would imply an annualized rate of approx. 2.68/10,000. While this hypothetical rate exceeds our calculated expected rate (2.2146/10,000), it still represents a flawed baseline. The structural error introduced by the mandatory exclusion of the high-risk <20 age stratum, a population known to have incidence rates up to four times higher [e.g.,3.4241/10,000 in certain strata, representing approximately 15% of the South Korean population (Kang and Lee, 2023)] fundamentally invalidates the IR in the control group. Any potential under- or over-reporting bias is secondary to this core methodological flaw, which makes the observed HR an unreliable measure.

Finally, it is crucial to emphasize how our robust, age-specific standardization analysis has shown that the structural age difference explains only 12.28% of the excess risk signaled by the authors of Kim et al. (2024). The overwhelming majority of the association 87.72% resulting in an HR Residual larger than 2.24 is entirely attributable to uncorrected methodological flaws and unmeasured confounding which should be attributed to a basic failure in the construction of the cohort and their subgroups as hypothesized below.

### 3.2 Recalculation of risk: the collapse of the observed association

The massive quantitative discrepancy identified in Section 3.1, where the IR (NV, Observed) of 0.67/10,000 falls short of the IR (NV, Expected) of 2.2146/10,000, by nearly 70%, due to non-comparability bias, allows us to perform a fundamental recalculation of risk.

We perform this calculation as a necessary application of risk decomposition methodology. Given the established 70% quantitative failure of the observed denominator IR (NV, Observed), this observed rate is deemed fundamentally unreliable and invalid for any comparative calculation. Therefore, the only methodologically sound baseline for calculating a *plausible* risk ratio is to replace the flawed observed denominator with the externally validated, demographically expected national baseline IR (NV, Expected) calculated in the presious sections following the principles of HR decomposition inspired by the theory developed in Oaxaca (1973), Blinder (1973).

We hypothesize that had the NV cohort been properly matched for unmeasured confounders, the true baseline risk would have approached the expected incidence rate of 2.2146/10,000. We recognize that this assumption, that IR (NV, Expected) represents the least biased baseline, still carries a risk of residual confounding (Type II bias). However, we maintain this assumption is methodologically superior to the observed rate [IR (NV, Observed)], which is proven to be profoundly flawed by a 70% deficit due to selection and detection biases. Assuming, for the sake of this demonstration, that the Detection Bias acting on the Vaccinated (V) cohort is negligible (i.e., that IR (V, Observed) is approximately equal to IR (V, True), we can calculate the resulting Hazard Ratio Hr Corrected as follows: HR Corrected = IR (V, Observed)/IR (NV, Expected), yielding \approximately 1.0025.

To notice is the fact that the purpose of this calculation is not to establish a definitive, unbiased risk estimate, but to demonstrate that *even if* the numerator (V cohort) is correct, the spurious signal collapses to a HR of almost 1 once the proven methodological error in the denominator is neutralized. This approach isolates the contribution of the invalid denominator as the primary quantitative driver of the original HR = 2.714.

This simple recalculation demonstrates that by correcting the methodological failure of the denominator alone, the observed risk signal of 2.714 collapses entirely to ≈1. An HR of 1 would mean a clinically insignificant increased risk, effectively reducing the study’s finding to a null hypothesis.

## 4 Discussion

We will summarize here the key takeaways of this discussion into two primary issues to ensure they are properly highlighted, noting that at the heart of the matter lie problems of loss of comparability and resulting clinical significance.

### 4.1 The collapse of internal validity: the double distortion mechanism

The persistence of the high residual 2.2423 after robust adjustment for age structure HR Structural = 1.2104 provides a definitive quantitative proof that the cohorts have been constructed as non-comparable. The study’s design of Kim et al. (2024) suffers from a double distortion mechanism that fundamentally violates the core premise of observational epidemiology.

First, the artificial baseline depression of the NV sub-group is attributed to the Selection Bias revealed in Section 3.1: the IR (NV) being 70% lower than expected indicates that the individuals composing the denominator are not representative of the general population. This massive deficit is caused by the inclusion of a minority of older individuals ( ≥ 60 years) who are part of an exceptionally healthy survivor cohort and/or have minimal medical interaction (Chemaitelly et al., 2025), thus ensuring profound under-detection of Vitiligo cases. This failure to capture the true baseline risk, depressing the denominator from the expected of approx. 2.21/10,000 to the observed 0.67/10,000, is the primary quantitative driver of the resulting high HR Residual ≈2.24.

Second, we confront an inflated incidence by detection and risk in the V subgroup. Conversely, the V group’s composition of Kim et al. (2024) (≈70% aged ≥ 50 years) guarantees maximal risk exposure because this demographic actively intercepts the second, higher incidence peak of Vitiligo (as seen in the Incidence Rate data before). Notably, the heavy representation in the (50–59) year age stratum (24.76% of the V cohort vs. 14.16% of the NV cohort) positions the V group directly into the initial phase of this second peak, maximizing both the intrinsic risk and the diagnostic detection rate. This structural flaw, coupled with the heightened surveillance and utilization bias typical of vaccinated groups, ensures that both the intrinsic risk and the diagnostic detection rate are maximized. This extreme structural separation, especially in the high-risk and high-surveillance age categories, represents a violation of the common support assumption. The Cox model employed in the scrutinized study, therefore, did not compare like with like, but rather measured the risk differential between an artificially clean control group and a maximally surveilled risk group.

### 4.2 Clinical and external validity implications for vaccine safety verification

The structural bias quantified by our formal direct age-standardization analysis has profound and critical implications for both the clinical interpretation of the findings and the external validity of the scrutinized study (Kim et al., 2024). The true magnitude of the risk associated with vaccination is demonstrably found to be almost equal to 1, which signifies a negligible or clinically insignificant effect, drastically contrasting with the alarmist published figure of HR = 2.714. This powerful evidence strongly suggests that the reported association is a spurious signal, driven predominantly by the systematic demographic differences and the resulting non-comparability bias between the intervention and reference cohorts, rather than by a genuine, underlying biological effect of the vaccine. This pervasive overestimation of risk not only undermines scientific integrity but can lead to misguided clinical counseling, unnecessary patient anxiety, and the misallocation of precious public health resources.

With this respect, structurally biased findings pose a severe threat to post-marketing pharmacovigilance systems by consistently generating false-positive safety signals. The uncorrected 2.714 value, if accepted without critical scrutiny, demands immediate regulatory attention, potentially leading to unnecessary policy interventions and the inevitable erosion of public trust in vaccine safety monitoring. We contend that the extreme quantitative evidence of non-comparability we have demonstrated, the nearly 70% failure in the baseline rate, represents a clear violation of core methodological principles, including those enshrined in the STROBE statement (Elm et al., 2007). The visible and substantial deviations from expected national baseline incidence should have obligated the original authors, under the guidelines of STROBE Article 12 (Discussion), to provide a robust, transparent, and extensive discussion on the reasons for these baseline deviations and the potential impact of such severe structural biases on the final reported HR value. To effectively prevent such profound methodological failures from entering the public domain and subsequently influencing policy, we strongly recommend that regulatory bodies mandate specific, quantitative safeguards for all observational studies utilizing non-matched retrospective cohorts. These essential safeguards should explicitly include: a) *Quantitative Baseline Validation:* Mandating a systematic comparison between the observed crude incidence rate IR (Observed) in the reference group and the expected rate IR (Expected) derived from demographically weighted national data. 2; b) *Mandatory Common Support Demonstration*: Requiring proof that the intervention and reference cohorts share a sufficiently overlapping distribution of key confounders, or that an appropriate statistical method (e.g., Propensity Score Matching) was rigorously used and validated to achieve common support before any risk estimate is calculated. We reiterate that this methodological failure is a serious breach of epidemiological reporting standards in the sense of the STROBE protocol and undermines the utility of national registry data for assessing vaccine safety signals when proper cohort matching is neglected.

Not only that but while our specific analysis utilizes epidemiological data anchored to the South Korean population and Vitiligo, the direct age-standardization framework developed herein is universally applicable to quantify structural bias in any observational study context globally. Provided that reliable, stratified external incidence data is available for the condition and population under study, this framework offers a transparent, efficient, and methodologically rigorous tool to empirically test the fundamental assumption of baseline comparability, an assumption that is foundational to the validity of all non-randomized risk analyses.

### 4.3 Limitations and future directions

We acknowledge several limitations to our direct age-standardization analysis. First, the HR Structural calculation relies on the assumption that the external, age-specific incidence rates IR(i) derived from the general South Korean population (as reported from different perspectives in all the available literature [(Akl et al., 2024; Kang and Lee, 2023; Lee et al., 2015)] accurately reflect the true baseline risk within the national health insurance service data utilized in Kim et al. (2024).

We acknowledge the omission of 95% Confidence Intervals for HR Structural, HR Residual, and HR Corrected. Yet this has a mathematical motivation. These metrics in fact are derived deterministically by combining a published point estimate HR Observed of Kim et al. (2024) with weighted mean age-specific incidence rates from external literature. As these are ratios of weighted means, they do not produce a variance estimate suitable for standard CI calculation methods (such as those used in regression models). We maintain that the point estimates are robust quantitative measures of the bias, and the primary conclusion, that the signal collapses from 2.714 to almost unity upon denominator correction, is highly stable.

Second, our analysis only addresses confounding introduced by structural age differences; we are unable to quantify the residual contributions of other unmeasured variables, such as socioeconomic status (SES), co-morbidities, or the precise effect of Detection Bias related to varying healthcare utilization frequency, all of which likely inflated the HR Residual. We obviously acknowledge that this study is authored by a single researcher. However, the author possesses over 20 years of experience in theoretical and applied statistics and biostatistics, including quantitative analysis in medical contexts. The methodologies employed (Direct Age-Standardization and multiplicative risk decomposition) are grounded in established, verifiable principles of classical epidemiology and biostatistics, which are fully and transparently presented herein. Therefore, the methodological rigor is maintained by strict adherence to statistical best practices, reinforcing the independent nature of this validation analysis. Furthermore, this methodological criticism does not exclude the possibility of a smaller, genuine biological signal HR < 1.2104), which would be revealed only through a properly designed study utilizing tight Propensity Score Matching and time-varying exposure analysis which was clearly not used by the authors of Kim et al. (2024).

Nonetheless, we maintain that our present analysis has provided a crucial quantitative framework for critically assessing the validity of large epidemiological risk estimates derived from imbalanced cohorts and providing a relevant contribution towards the fidelity and verifiability of vaccine safety signals derived from observational cohort data.

## 5 Conclusion

The association between COVID-19 vaccination and Vitiligo (HR = 2.714) as reported in Kim et al. (2024) is an extreme statistical artifact. Our robust direct age-standardization analysis, based on specific South Korean age-incidence rates, definitively proves that the structural age imbalance explains only a minor fraction (HR Structural ≈1.21) of the observed risk. The overwhelming HR Residual of 2.24 is the quantitative measure of the methodological failure and residual confounding caused by the Structural Selection and Detection Bias that have affected the construction of the retrospective cohort of Kim et al. (2024). This failure to establish genuinely comparable cohorts has compromised both the internal validity and external validity of the investigated study. The reported finding of Kim et al. (2024) is therefore an association primarily driven by bias (a spurious association) and should not be used to inform public health policy or safety communication. Our attempt to recalculate the real risk demonstrates that correcting the denominator’s failure results in an HR ≈ 1, which is a plausible outcome consistent with the fundamental rules of biostatistics required for a valid cohort study. However, while the overwhelming signal is artifactual, only a methodologically sound study could definitively determine if a small, genuine biological signal persists. We conclude by urging the re-evaluation and potential reconsideration of the study’s conclusions. Our findings also underscore the persistent reliance on and respect for methodological goldstandards in clinical research (Roccetti and Cacciapuoti, 2025; Jones and Podolsky, 2015). While innovation in epidemiological design is crucial, these established benchmarks must only be challenged or superseded by new studies featuring superior internal validity and robust correction for all known sources of bias.

### 5.1 Demonstration of clinical urgency

Our study stems from the need to counteract the threat to clinical integrity posed by a flawed finding indicating major risk for Vitiligo after Covid-19 vaccination. This spurious signal is misleading for patients, clinicians, and health authorities. By demonstrating the complete collapse of the risk signal after correct recalculation, we provide evidence neutralizing an artifactual safety concern.

### 5.2 Bias decomposition

We introduce a Stratified Ponderation Analysis that moves beyond simple confounding acknowledgement. We decompose the initial Hazard Ratio (HR) into two necessary components: the Structural HR which is the risk attributable to demographic imbalance, and the Residual HR which serves as a direct measure of all uncorrected design failures.

### 5.3 Proof of denominator Invalidity

Our investigation quantifies that the incidence rate in the reference cohort (Non-Vaccinated) is approximately 70% lower than the rate expected for its age structure. This provides evidence of failure in the control cohort, constituting a critical violation of common STROBE principles.

### 5.4 Correction of spurious risk

We demonstrate, through recalculation of risk, that the original, amplified HR collapses to a clinically irrelevant value. This outcome has a direct and urgent impact on vaccine safety verification.

## Data Availability

The original contributions presented in the study are included in the article/supplementary material, further inquiries can be directed to the corresponding author.
